# Long non-coding RNA MEG3 knockdown represses airway smooth muscle cells proliferation and migration via sponging miR-143-3p/FGF9 in asthma

**DOI:** 10.1186/s13019-024-02798-5

**Published:** 2024-06-01

**Authors:** Jiaying Gu, Dengfeng Zhou

**Affiliations:** Department of Pulmonary and Critical Care Medicine, Wuhan Fourth Hospital, No. 76 Jiefang Avenue, Qiaokou District, Wuhan, 430000 China

**Keywords:** Airway smooth muscle cells, lncRNA MEG3, miR-143-3p/FGF9 axis, Asthma, Proliferation, Migration

## Abstract

**Background:**

Asthma is a respiratory disease characterized by airway remodeling. We aimed to find out the role and mechanism of lncRNA MEG3 in asthma.

**Methods:**

We established a cellular model of asthma by inducing human airway smooth muscle cells (HASMCs) with PDGF-BB, and detected levels of lncRNA MEG3, miR-143-3p and FGF9 in HASMCs through qRT-PCR. The functions of lncRNA MEG3 or miR-143-3p on HASMCs were explored by cell transfection. The binding sites of miR-143-3p and FGF9 were subsequently analyzed with bioinformatics software, and validated with dual-luciferase reporter assay. MTT, 5-Ethynyl-2’-deoxyuridine (EdU) assay, and Transwell were used to detect the effects of lncRNA MEG3 or miR-143-3p on proliferation and migration of HASMCs. QRT-PCR and western blot assay were used to evaluate the level of proliferation-related marker PCNA in HASMCs.

**Results:**

The study found that lncRNA MEG3 negatively correlated with miR-143-3p, and miR-143-3p could directly target with FGF9. Silence of lncRNA MEG3 can suppress migration and proliferation of PDGF-BB-induced HASMCs via increasing miR-143-3p. Further mechanistic studies revealed that miR-143-3p negatively regulated FGF9 expression in HASMCs. MiR-143-3p could inhibit PDGF-BB-induced HASMCs migration and proliferation through downregulating FGF9.

**Conclusion:**

LncRNA MEG3 silencing could inhibit the migration and proliferation of HASMCs through regulating miR-143-3p/FGF9 signaling axis. These results imply that lncRNA MEG3 plays a protective role against asthma.

## Introduction

Asthma is the chronic respiratory disease affecting children and adults, and is characterized by inflammation of the bronchi, increased viscous secretions within the tubes, and airway remodeling [1–3]. Clinically, asthma is manifested as cough, wheezing, chest tightness, etc., which can cause lung infection and sudden death in severe cases [3]. There is currently no cure for asthma, and treatment just could control the symptoms. For example, studies have found that anti-inflammatory drugs and bronchodilators can be effective in controlling asthma [4–6]. However, traditional medications are associated with a variety of side effects and exploring new treatments is expected to be the key to treating asthma.

Accumulating evidences suggested abnormal migration and proliferation of HASMCs were responsible for changes in ASM thickness and promoting airway remodeling [7–9]. In addition, the mechanism of airway remodeling is related to the inflammatory-promoting factors and growth factors released by inflammatory cells in the airway [10, 11]. In the presence of a variety of inflammatory factors, the airway epithelium in asthmatics undergoes pathological changes, such as basement membrane thickening, glandular hypertrophy and hypertrophy of smooth muscle [12]. The study found that the expression of platelet-derived growth factor BB (PDGF-BB) was obviously increased on asthmatic tissues [13]. Moreover, PDGF-BB can induce proliferation and migration of HASMCs, causing airway remodeling [14, 15]. Therefore, inhibiting proliferation and migration of PDGF-BB-stimulated HASMCs can effectively prevent occurrence of airway remodeling, which may become an effective treatment for asthma.

Long non-coding RNAs (lncRNAs) are a type of RNAs do not encode proteins and have transcripts longer than 200 nt [16, 17]. LncRNAs have multiple functions, for instance acting as molecular scaffolds in the nucleus, regulating chromosome structure, or as competing endogenous RNAs (ceRNAs) in cytoplasm to promote or inhibit mRNA degradation and adsorb microRNAs (miRNAs) [18, 19]. In addition, lncRNAs participated in regulation of various biophysiological processes, including embryonic development, cardiac development, the immune system, and the endocrine system [20–22]. Aberrant level of lncRNAs has been found in various of diseases, such as cardiovascular diseases, tumours and neurodegenerative diseases [23–26]. Thus, measuring the expression of lncRNAs in different cells or diseases could help to understand their function, or to identify valid molecular markers. Recent studies have shown thatthe expression of lncRNA Maternally-Expressed Gene 3 (MEG3) was significantly reduced in the peripheral blood of asthma patients [27], and lncRNA MEG3 played important roles in asthma by regulating Treg/Th17 balance [28]. The results indicate that lncRNA MEG3 is involved in the mechanism of asthma, while the specific roles and regulatory mechanisms remain to be further investigated.

Increasing evidences demonstrate lncRNAs could act as ceRNAs to regulate the role of miRNAs in disease [18, 29]. MiRNA is a class of non-coding RNAs with a length of 21–23 nucleotides, which regulating gene expression at the translational level [30, 31]. Research have shown miR-143-3p is the direct target of lncRNA MEG3, which could be involved in periodontitis by sponging miR-143-3p to inhibit AKT/IKK signaling pathway [32]. MiR-143-3p is a tumor suppressor that affects invasion, migration and proliferation [33–35]. MiR-143-3p was found to be obviously decreased in ASMCs of asthmatic patients and may exert a protective effect by inhibiting asthmatic airway remodeling [36]. However, it is unclear whether lncRNA MEG3 plays an important role in asthma through regulating miR-143-3p.

This study aimed to explore the roles and underlying mechanism of lncRNA MEG3 in proliferation and migration of ASMCs.

## Materials and methods

### Cell culture and drug treatment

We purchased human airway smooth muscle cells (HASMCs) from Shang Hai Ze Ye Biotechnology Co., Ltd. (Cat. no. AC339826, Shanghai, China), and cultured with high glucose-DMEM (BasalMedia, Shanghai) medium containing 10% fetal bovine serum (FBS, Biological Industries). HASMCs were placed in a 37 °C incubator with 5% CO_2_.

Previous studies have found PDGF-BB could induce airway remodeling in asthma [15]. A cellular model of asthma was set up through stimulating HASMCs with PDGF-BB (25 ng/ml; Sigma, USA) for 24 h in this study.

### Cell transfection

MEG3-siRNA and control-siRNA were synthesized from GenePharma, and FGF9 plasmid (sc-403,118-ACT) and control plasmid (sc-437,275) were obtained from Santa Cruz Biotechnology. The miR-143-3p inhibitor and mimic were obtained from ThermoFisher. SiRNAs, plasmids, miR-143-3p mimic or inhibitor were transfected into HASMCs using Lipofectamine™ 2000 (Invitrogen) according to manufacturer’s instruction. Using qRT-PCR to test transfection efficiency after 48 h.

### RNA extraction and quantitative RT-PCR (qRT-PCR)

We extracted total RNA from HASMCs with RNA-easy Isolation Reagent (R701-01, Vazyme), and then cDNA was obtained with HiScript II Q RT SuperMix (R222-01, Vazyme). The qRT-PCR was subsequently performed with AceQ qPCR SYBR Green Master Mix (Q111-02, Vazyme). Primers were synthesized from Sangon Biotech (Shanghai, China) with following sequences: lncRNA MEG3 F: 5’- GCTCTACTCCGTGGAAGCAC-3’, R: 5’-CAAACCAGGAAGGAGACGAG-3’; miR-143-3p F: 5’-GGGGTGAGATGAAGCACTG-3’, R: 5’-CAGTGCGTGTCGTGGAGT-3’; PCNA F: 5’-CTAGCCATGGG CGTGAAC-3’, R: 5’-GAATACTAGTGCTAAGGTGTCTGCAT-3’; FGF9 F: 5’-GCAGTCACGG ACTTGGATCAT-3’, R: 5’-TTCTCGTTCATGCCGAGGTAG-3’; GAPDH F: 5’-CGGAGTCAACGGATTTGGTCGTAT-3’, R: 5’-AGCCTTCTCCAT GGTGGTGAAGAC-3’; U6 F: 5’-CTCGCTTCGGCAGCACATATACT-3’, R: 5’-ACGCTTCACGAATTTGCGTGTC-3’.

### Western blot assay

Total protein was obtained with RIPA lysis buffer (P0013B, Beyotime, Shanghai, China). The supernatant was collected through centrifugation at 12,000 rpm for 5 min, and the total protein was detected by a BCA kit (P0009, Beyotime, Shanghai, China). 30 µg total proteins were subjected to SDS-PAGE, and transferred to PVDF membranes (Millipore). Membranes were then blocked with 5% skimmed milk for 1 h. Then, primary antibodies including anti-PCNA (ab92552, 1: 3000, Abcam), anti-FGF9 (ab206408, 1: 1000, Abcam), and anti-GAPDH (ab181602, 1: 10,000, Abcam) were incubated overnight at 4 °C. The next day, membranes were washed 5 times with TBST buffer for 5 min each time. Membranes were then incubated with HRP-labeled secondary antibody (AS1107, 1: 10,000, ASPEN). After 2 h, bands were displayed with ECL luminescent solution.

### MTT assay for cell viability

Proliferation was measured with MTT assay as previously described [37]. Briefly, seeding 1 × 10^4^ cells into a 96-well plate and treated for 24 h before MTT assay. Adding 20 µl MTT solution (C0009S, Beyotime, Shanghai, China) to each well and cells were cultured for 4 h at 37 °C. Absorbance levels were measured at 570 nm with a plate reading spectrophotometer.

### 5-Ethynyl-2’-deoxyuridine assay

*5-Ethynyl-2’-deoxyuridine* (EdU) assay was performed to assess cell proliferation. Briefly, HASMCs were seeded in 96-well plates and then treated with 50 µM EdU solution (C10310-1, RIBOBIO) for 2 h. The cells were fixed with 2% paraformaldehyde for 20 min and stained with Apollo staining reaction solution (C10310-1, RIBOBIO) at room temperature in dark for 10 min. Finally, cells were analyzed using a FACSCalibur flow cytometer (Beckman).

### Transwell assay for migration

Migration was assessed with Transwell assay as previously described [38]. Briefly, serum-free and 10% serum-containing medium were added to the upper and lower layers in a 24-well Transwell chamber with a pore size of 8 μm. Cells were cultured in the upper layer for 24 h. Cells penetrating to the lower layer were subsequently fixed with 4% methanol and stained with crystal violet. Cells were counted with an inverted microscope (LEICA).

### Dual-luciferase reporter gene assay

TargetScan 7.2 database (http://www.targetscan.org/vert_72/) was used to predict binding sites between miR-143-3p and FGF9. Subsequently, dual-luciferase reporter gene assay was performed as previously described to verify the relationship [39] Briefly, mimic control, miR-143-3p mimic, FGF9-3’UTR-WT and FGF9-3’UTR-MUT were synthesized from Sangon Biotech (Shanghai, China). FGF9-3’UTR-WT or FGF9-3’UTR-MUT were cotransfected with miR-143-3p mimics or mimic control using Lipofectamine™ 2000 (Invitrogen). After 48 h, luciferase activity was analyzed with dual luciferase reporter assay system (Promega).

### Statistical analysis

Data were statistically analyzed with GraphPad Prism 9 and were expressed as mean ± standard deviation (SD). Unpaired T-test or one-way ANOVA was used for analysis, and *p* < 0.05 indicated statistical significance.

## Results

### LncRNA MEG3 was upregulated, whereas mir-143-3p was downregulated in PDGF-BB-induced HASMCs

**Fig. 1:**
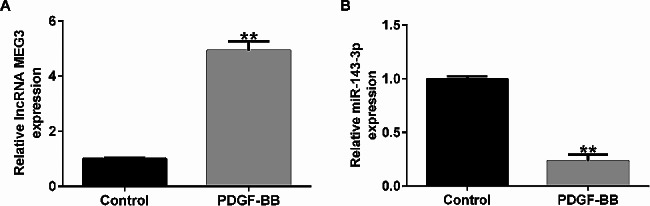
Expression of lncRNA MEG3 and miR-143-3p in PDGF-BB-induced HASMCs. (**A**-**B**). The expression of lncRNA MEG3 (**A**) and miR-143-3p (**B**) were detected by qRT-PCR. **p < 0.01 vs. control group

To explore roles of lncRNA MEG3 and miR-143-3p in asthma, this study constructed an asthma model by treating HASMCs with PDGF-BB, and detected the levels of lncRNA MEG3 and miR-143-3p with qRT-PCR. Compared with control group, lncRNA MEG3 was remarkably upregulated in PDGF-BB-induced HASMCs, while miR-143-3p was significantly reduced in PDGF-BB-induced HASMCs (Fig. 1A and B). These results suggest lncRNA MEG3 and miR-143-3p may be involved in development of asthma.

### LncRNA MEG3 negatively regulates mir-143-3p in HASMCs

**Fig. 2:**
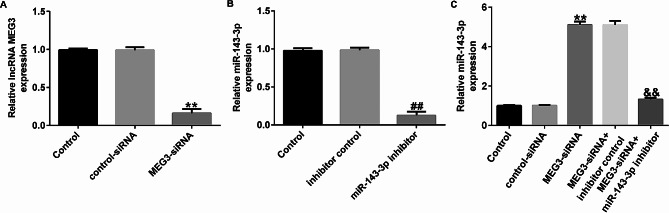
LncRNA MEG3 negatively regulates miR-143-3p in HASMCs. (**A**-**C**). The expression of lncRNA MEG3 and miR-143-3p were detected with qRT-PCR. **P < 0.01 vs. Control-siRNA; ##*P* < 0.01 vs. Inhibitor control; &&*P* < 0.01 vs. MEG3-siRNA + inhibitor control

Then, we investigated the relationship between lncRNA MEG3 and miR-143-3p in asthma by transfecting control-siRNA, MEG3-siRNA, inhibitor control, miR-143-3p inhibitor, MEG3-siRNA + inhibitor control, and MEG3-siRNA + miR-143-3p inhibitor to HASMCs. Using qRT-PCR to examine the transfection efficiency after 48 h. The results implied MEG3-siRNA could reduce the expression of lncRNA MEG3 (Fig. 2A), while miR-143-3p inhibitor significantly reduced the expression of miR-143-3p (Fig. 2B). Furthermore, MEG3-siRNA significantly enhanced the level of miR-143-3p in HASMCs compared with control-siRNA group, and this effect could be significantly reversed by co-transfection with miR-143-3p inhibitor (Fig. 2C). These results prove that lncRNA MEG3 is negatively correlated with miR-143-3p in HASMCs.

### LncRNA MEG3-siRNA significantly inhibits proliferation and migration of PDGF-BB-induced HASMCs by upregulating miR-143-3p

**Fig. 3:**
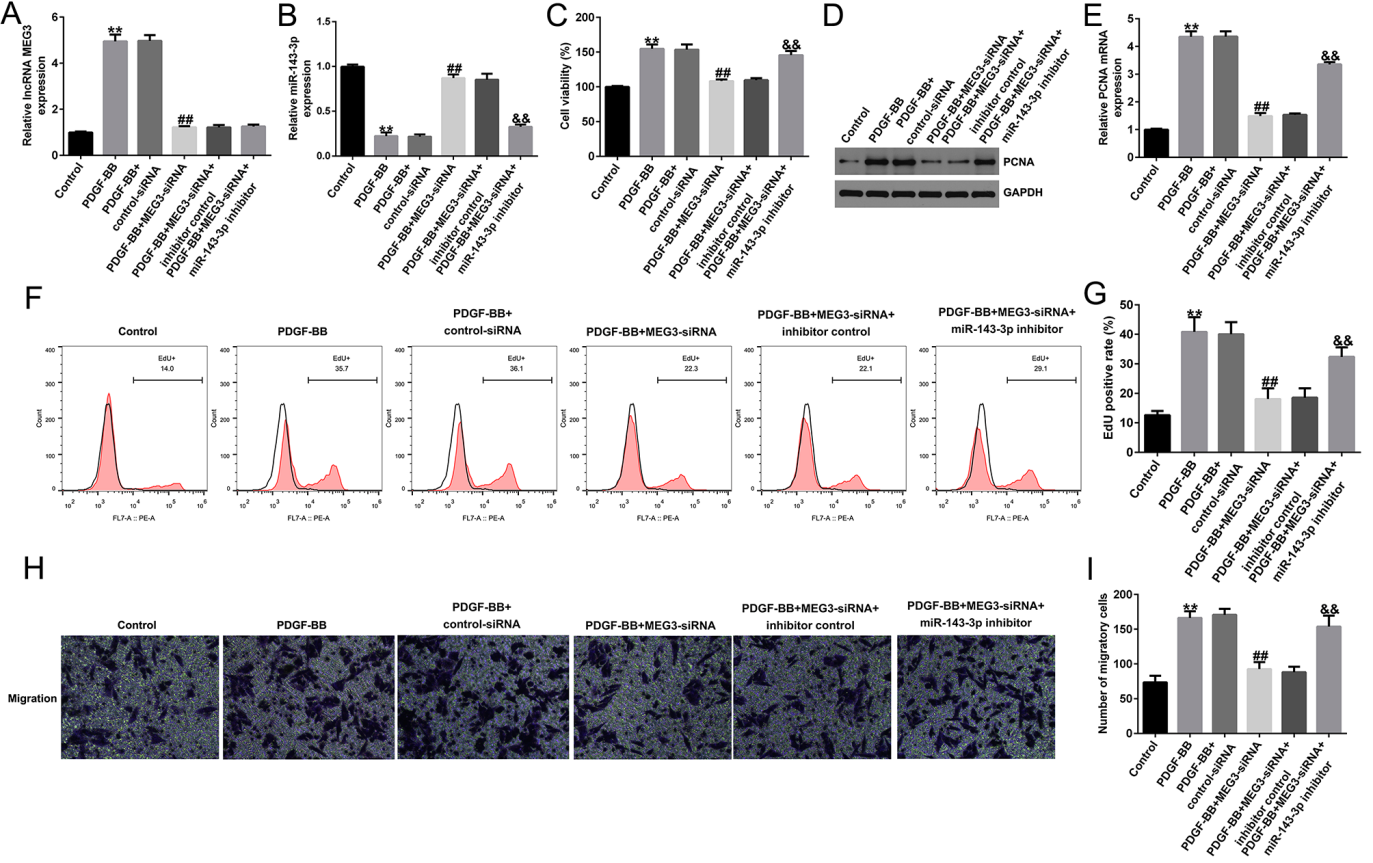
Silencing of lncRNA MEG3 inhibits the proliferation and migration of PDGF-BB-induced HASMCs by upregulating miR-143-3p. (**A**-**B**). The expression of lncRNA MEG3 and miR-143-3p were detected by qRT-PCR; (**C**). MTT assays were used to evaluate the cell proliferation of HASMCs; (**D**-**E**). Western blotting and qRT-PCR were used to analyze the protein (**D**) and mRNA (**E**) levels of PCNA; (**F**). EdU assay for cell proliferation detection; (**G**). EdU positive rate; (**H**) and (**I**). Transwell assays were used to detect the migration of HASMCs. **p < 0.01 vs. Control group; ##*p* < 0.01 vs. PDGF-BB + control-siRNA group; &&*p* < 0.01 vs. PDGF-BB + MEG3-siRNA + inhibitor control group

To explore whether miR-143-3p regulates role of lncRNA MEG3 in asthma, we performed loss-of-function experiments. First, we transfected control-siRNA or MEG3-siRNA into HASMCs, and co-transfected inhibitor control or miR-143-3p inhibitor and MEG3-siRNA into HASMCs. After 48 h, using 25 ng/ml PDGF-BB to induce cells for 24 h. In PDGF-BB-induced HASMCs, the expression of lncRNA MEG3 was increased, while the expression of miR-143-3p was significantly decreased (Fig. 3A and B). In addition, MEG3 knockdown significantly reduced the level of MEG3 in PDGF-BB-stimulated HASMCs, while miR-143-3p was significantly increased (Fig. 3A and B). However, cotransfection of miR-143-3p inhibitor with MEG3-siRNA could reverse this effect (Fig. 3A and B).

Subsequently, cell proliferation was measured with MTT and EdU assay, cell migration was detected with Transwell, and expression of cell proliferation related gene PCNA was detected by western blot and qRT-PCR. The results revealed cell viability of HASMCs induced by PDGF-BB was significantly increased (Fig. 3C), the protein and mRNA levels of PCNA was significantly increased (Fig. 3D-E), the prolifearion of HASMCs was signifixantly enhanced (Fig. 3F anf G) and the cell migration ability was significantly improved (Fig. 3H and I). In addition, in PDGF-BB-induced HASMCs, MEG3 downregulation significantly reduced cell proliferation and cell migration, and PCNA was reduced (Fig. 3C-I). However, these changes were significantly reversed via cotransfection with miR-143-3p inhibitor. These results suggest downregulation of lncRNA MEG3 can inhibit proliferation and migration of PDGF-BB-induced HASMCs by upregulating miR-143-3p.

### MiR-143-3p directly targets FGF9

**Fig. 4:**
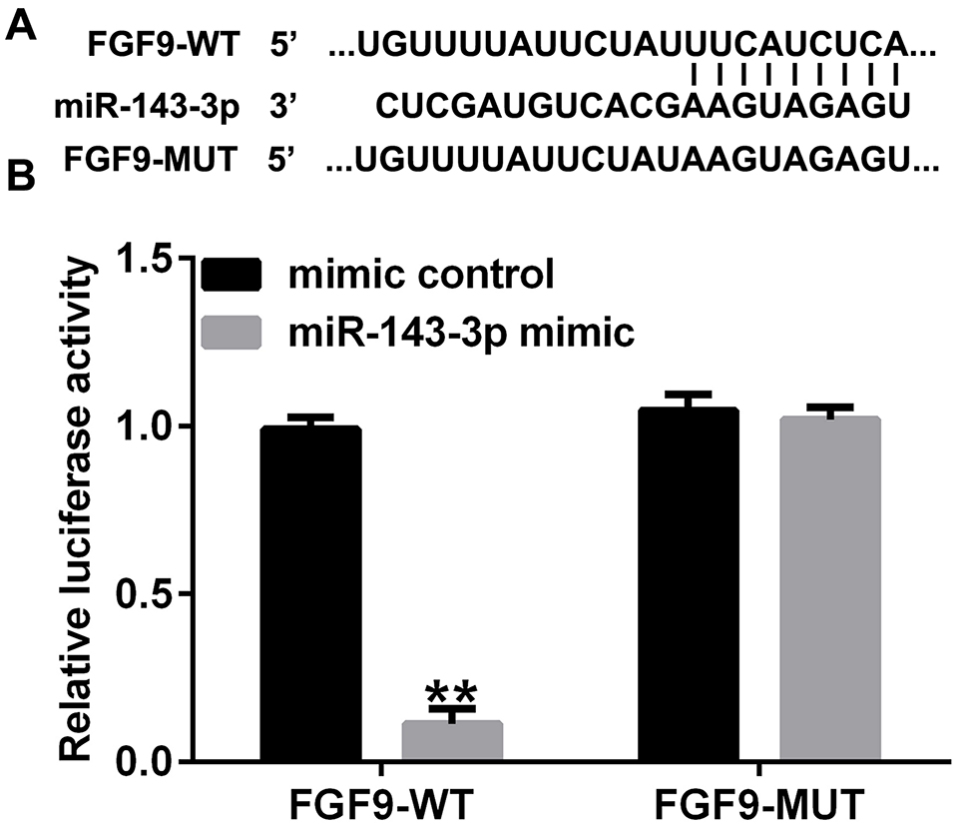
Determination of the relationship between miR-143-3p and FGF9. (**A**). TargetScan predicted the binding sites between miR-143-3p and FGF9; (**B**). The interaction between miR-143-3p and FGF9 is verified by dual-luciferase reporter assay. **p < 0.01 vs. mimic control

To investigate mechanisms of miR-143-3p in proliferation and migration of PDGF-BB-induced HASMCs, we used TargetScan to predict the targets of miR-143-3p. The results indicated miR-143-3p had binding sites with FGF9 (Fig. 4A). Subsequently, we used dual-luciferase reporter gene assay to verify the relationship between miR-143-3p and FGF9. FGF9-3’UTR-WT or FGF9-3’UTR-MUT were co-transfected into HASMCs with miR-143-3p mimic or control mimic. As shown in Fig. 4B, miR-143-3p mimic could specifically reduce luciferase activity of FGF9-3’UTR-WT, but had no effect on luciferase activity of MUT. The data suggests FGF9 is a direct target of miR-143-3p.

### Expression of FGF9 in PDGF-BB-induced HASMCs

**Fig. 5:**
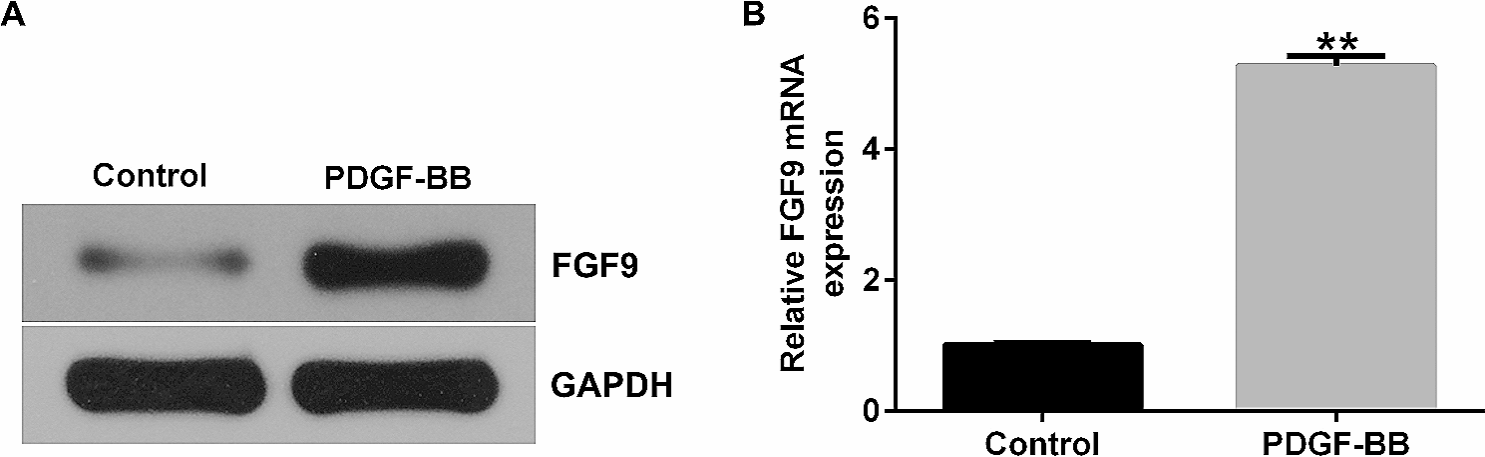
Expression of FGF9 in HASMCs. (**A**-**B**). the protein (**A**) and mRNA (**B**) levels of FGF9 were detected by Western blotting and qRT-PCR. **p < 0.01 vs. Control

Next, we examined the levels of FGF9 in PDGF-BB-stimulated HASMCs with western blot assay and qRT-PCR. The results demonstrated that the protein and mRNA levels of FGF9 were obviously increased in PDGF-BB-induced HASMCs compared to control group (Fig. 5A and B). These results demonstrate that the expression of FGF9 is elevated in PDGF-BB-stimulated HASMCs.

### MiR-143-3p negatively regulates FGF9 in HASMCs

**Fig. 6:**
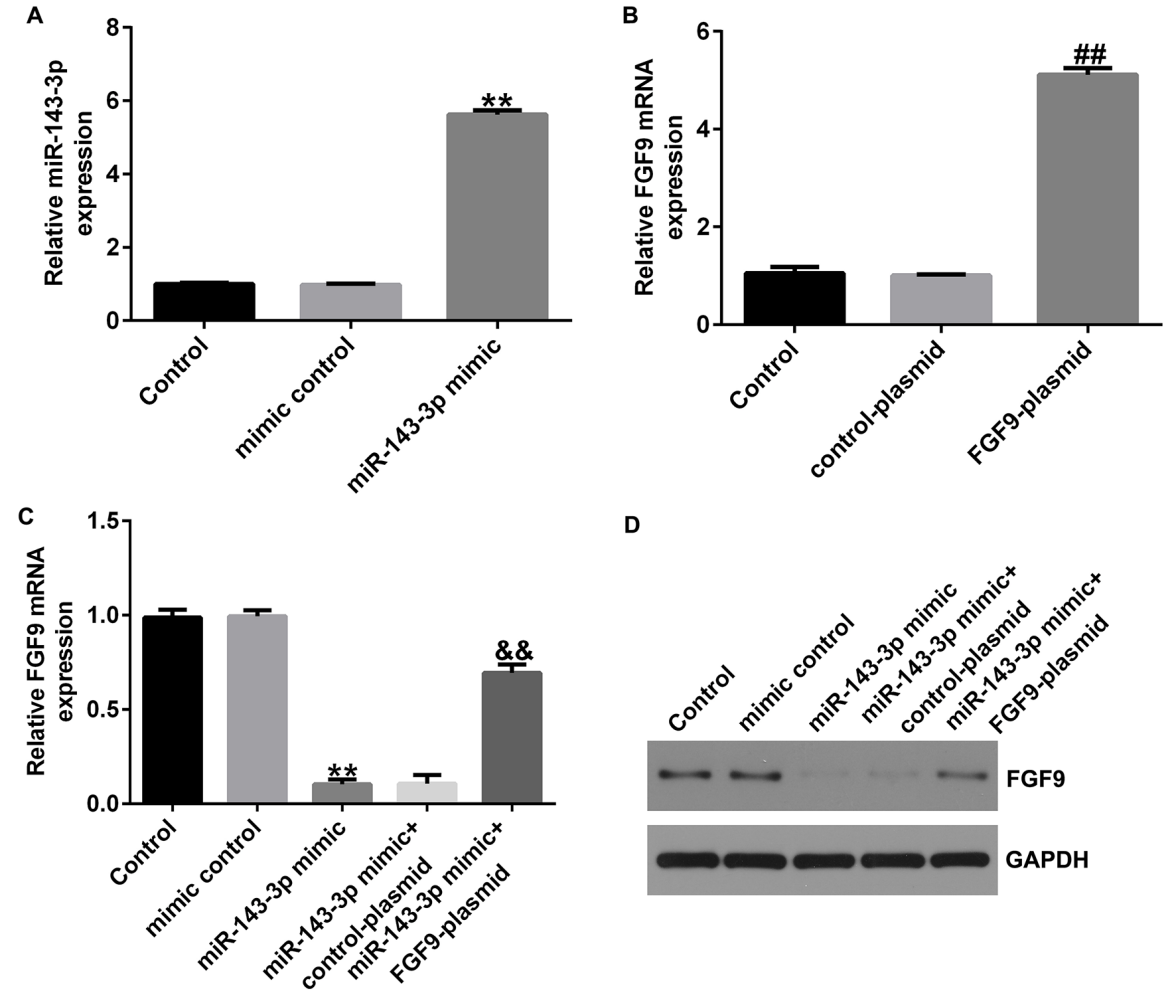
MiR-143-3p negatively regulates FGF9 in HASMCs. (**A**-**C**). The expression of miR-143-3p and FGF9 were detected by RT-qPCR. (**D**). The protein expression of FGF9 were detected by western blot assay. **P < 0.01 vs. Mimic control; ##*P* < 0.01 vs. Control-plasmid; &&*P* < 0.01 vs. miR-143-3p mimic + control-plasmid

To investigate relationship between miR-143-3p and FGF9, we transfected mimic control, miR-143-3p mimic, control-plasmid or FGF9-plasmid into HASMCs, and control-plasmid or FGF9-plasmid was co-transfected with miR-143-3p mimic into HASMCs. Results of qRT-PCR showed miR-143-3p mimic significantly increased miR-143-3p (Fig. 6A), and FGF9-plasmid significantly increased FGF9 levels (Fig. 6B). Furthermore, upregulation of miR-143-3p could reduce the expression of FGF9 in HASMCs, which was reversed by co-transfection of miR-143-3p mimic with FGF9-plasmid (Fig. 6C and D). These results suggest that miR-143-3p can negatively regulate FGF9 in HASMCs.

### MiR-143-3p mimic significantly inhibits proliferation and migration of PDGF-BB-induced HASMCs by reducing FGF9

**Fig. 7:**
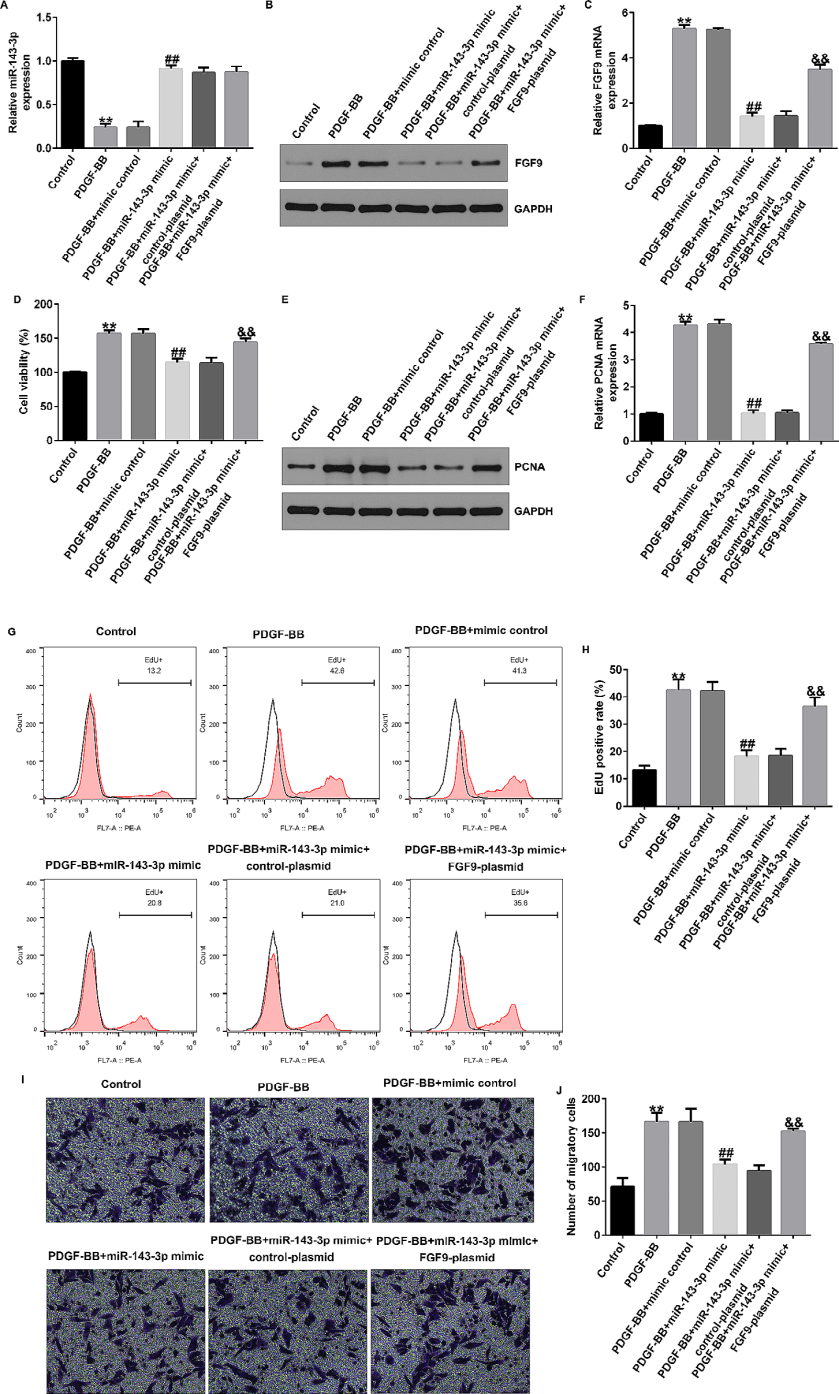
MiR-143-3p inhibits the proliferation and migration of PDGF-BB-induced HASMCs by downregulating FGF9. (**A**-**C**). The expression of miR-143-3p and FGF9 were detected by qRT-PCR and western blot assay; (**D**). MTT assays were used to evaluate the cell proliferation of HASMCs; (**E**-**F**). Western blotting and qRT-PCR were used to analyze the protein (**E**) and mRNA (**F**) levels of PCNA; (**G**). EdU assay for cell proliferation detection; (**H**) EdU positive rate; (**I**) and (**J**). Transwell assays were used to detect the migration of HASMCs. **p < 0.01 vs. Control group; ##*p* < 0.01 vs. PDGF-BB + mimic control group; &&*p* < 0.01 vs. miR-143-3p mimic + control-plasmid group

To further elucidate effects of miR-143-3p on proliferation and migration of PDGF-BB-induced HASMCs, we transfected mimic control or miR-143-3p mimic into HASMCs, and co-transfected control-plasmid or FGF9-plasmid with miR-143-3p mimic into HASMCs. After 48 h, using 25 ng/ml PDGF-BB to treat HASMCs for 24 h. Consistent with previous results, miR-143-3p was decreased in PDGF-BB-stimulated HASMCs, whereas FGF9 was significantly increased (Fig. 7A-C). Furthermore, upregulation of miR-143-3p significantly enhanced expression of miR-143-3p in PDGF-BB-stimulated HASMCs, whereas FGF9 was significantly decreased (Fig. 7A-C). However, the downregulation of FGF9 caused by miR-143-3p mimic was abolished by FGF9-plasmid (Fig. 7A-C).

MTT results showed PDGF-BB stimulation of HASMCs significantly increased cell viability, while upregulation of miR-143-3p decreased cell viability (Fig. 7D). Western blot and qRT-PCR results indicated the expression of PCNA in PDGF-BB group was significantly increased, while the upregulation of miR-143-3p could reduce the level of PCNA in PDGF-BB-induced HASMCs (Fig. 7E-F). The results of EdU assay indicated that PDGF-BB stimulation of HASMCs significantly enhanced cell proliferation, while upregulation of miR-143-3p decreased cell proliferation (Fig. 7G and H). Additionally, as shown in Fig. 7I and J, miR-143-3p mimic could significantly reduce cell migration ability of PDGF-BB-induced HASMCs. These effects were significantly reversed after co-transfection with FGF9-plasmid (Fig. 7D-J). These data suggest upregulation of miR-143-3p can significantly inhibit proliferation and migration by reducing FGF9.

## Discussion

Asthma is a common inflammatory disease of airways that usually results in airway remodeling [1, 9, 40]. Airway remodeling manifested by bronchial fibrosis, basement membrane thickening and increased airway smooth muscle mass [41]. The study found that abnormal proliferation and migration of ASMCs were involved in development of airway remodeling [7]. Therefore, modulating proliferation and migration of ASMCs may be an effective way to explore the therapy of asthma.

Evidences indicated lncRNA was the molecular marker for diagnosis of asthma and can be involved in regulating proliferation and migration of ASMCs [8, 42–44]. For example, downregulation of lncRNA MALAT1 inhibited proliferation of ASMCs through sponging microRNA-216a [45]. Recent years, lncRNA MEG3 was discovered to be a tumor suppressor gene, which can be involved in angiogenesis and tumorigenesis [46, 47]. LncRNA MEG3 was reported to affect proliferation and migration of prostate cancer cells through regulating miR-9-5p/QKI-5 axis [48]. Furthermore, lncRNA MEG3 could participate in neuronal apoptosis in stroke via sponging miR-424-5p [49]. Recent research have found lncRNA MEG3 was significantly increased in asthmatic patients [27], suggesting lncRNA MEG3 may be involved in the occurrence of asthma. The lncRNA MEG3 has been reported to regulate Treg/Th17 homeostasis in asthma patients through targeting microRNA-17 [28]. However, specific roles and regulatory mechanisms of lncRNA MEG3 in asthma is still unclear.

Recent studies have identified binding sites between lncRNA MEG3 and miR-143-3p, which was involved in regulating periodontal ligament cell damage [32]. However, the relationship between lncRNA MEG3 and miR-143-3p in asthma has not been reported. Our study resolved roles of lncRNA MEG3 and miR-143-3p in PDGF-BB-induced HASMCs. We proved lncRNA MEG3 could negatively regulate miR-143-3p, and silence of lncRNA MEG3 could suppress proliferation and migration of HASMCs via upregulating miR-143-3p. In addition, bioinformatics database indicated FGF9 was a direct target of miR-143-3p, and miR-143-3p was negatively correlated with FGF9. FGF9 is the member of the fibroblast growth factor family, which is involved in various pathological processes such as angiogenesis, apoptosis, and tumor growth [50]. FGF9 has been found to be essential for lung development and recovery from lung tissue injury [51]. Furthermore, FGF9 signaling inhibited airway smooth muscle differentiation in mouse lungs [52]. These studies imply that FGF9 may play important roles in smooth muscle cells. Our research showed miR-143-3p suppressed proliferation and migration of HASMCs through reducing the expression of FGF9, indicating the key role of FGF9 in the proliferation and migration of HASMCs in asthma. FGF9 may play an important role in asthma through regulating the proliferation and migration of HASMCs.

Our research is the first to elucidate the effects and mechanisms of lncRNA MEG3 and miR-143-3p on the proliferation and migration of HASMCs in airway remodeling in asthma. However, there were also some limitations of this study. Firstly, this study was mainly based on in vitro experiments to explore the role of lncRNA MEG3 in asthma, while in vivo studies need to be further validated by animal models. Besides, the expression of lncRNA MEG3/miR-143-3p/FGF9 in asthma patients and its correlation with clinical pathological parameters also need further clarification. This study also did not further explore the effects of FGF9 alone on HASMCs and on the protective effect of lncRNA MEG3 on asthma. In addition, lncRNA MEG3, as a ceRNA, can sponge a variety of miRNAs [53], and the involvement of lncRNA MEG3 in HASMCs function through regulation of miR-143-3p/FGF9 axis is one of the mechanisms identified in this study, while other mechanisms need to be explored through further studies. In future studies, we will further investigate these issues.

## Conclusions

In conclusion, our study revealed the roles of lncRNA MEG3 in PDGF-BB-induced HASMCs. Downregulation of lncRNA MEG3 could inhibit the proliferation and migration of HASMCs by regulating miR-143-3p/FGF9 signaling axis. These results suggest lncRNA MEG3 plays a protective role in asthma and may be a novel biomarker for therapy of asthma.

## Data Availability

The datasets used and/or analyzed during the present study are available from the corresponding author on reasonable request.
